# Surgical Techniques and Outcomes in Patients With Intra-Cardiac Abscesses Complicating Infective Endocarditis

**DOI:** 10.3389/fcvm.2022.875870

**Published:** 2022-05-31

**Authors:** Sam Straw, M. Wazir Baig, Vishal Mishra, Richard Gillott, Klaus K. Witte, Carin Van Doorn, Antonella Ferrara, Kalyana Javangula, Jonathan A. T. Sandoe

**Affiliations:** ^1^Faculty of Medicine and Health, University of Leeds, Leeds, United Kingdom; ^2^Department of Cardiology, Leeds Teaching Hospitals NHS Trust, Leeds, United Kingdom; ^3^Department of Cardiothoracic Surgery, Leeds Teaching Hospitals NHS Trust, Leeds, United Kingdom; ^4^Department of Microbiology, Leeds Teaching Hospitals NHS Trust, Leeds, United Kingdom

**Keywords:** infective endocarditis, abscess, aortic root abscess, cardiothoracic surgery, valve surgery

## Abstract

**Background:**

An intra-cardiac abscess is a serious complication of both native (NV-IE) and prosthetic valve infective endocarditis (PV-IE). Despite being an accepted indication for surgery, controversies remain regarding the optimal timing and type of operation. We aimed to report the outcomes of patients managed for intra-cardiac abscesses over more than a decade.

**Methods:**

Patients aged ≥18 years managed for intra-cardiac abscess between 1 January 2005 and 31 December 2017 were identified from a prospectively collected IE database. The primary outcome was 30-day mortality in operated patients and secondary outcomes were freedom from re-infection, re-operation and long-term mortality comparing those patients with aortic root abscess who underwent aortic valve replacement (AVR) and those who received aortic root replacement (ARR).

**Results:**

Fifty-nine patients developed an intra-cardiac abscess, and their median age was 55 (43–71) years; among them, 44 (75%) were men, and 10 (17%) were persons who injected drugs. Infection with beta-haemolytic streptococci was associated with NV-IE (*p* = 0.009) and coagulase-negative staphylococci with PV-IE (*p* = 0.005). Forty-four (75%) underwent an operation, and among those with aortic root abscess, 27 underwent AVR and 12 ARR. Thirty-day mortality was associated with infection with *S. aureus* (*p* = 0.006) but not the type or timing of the operation. Survival in operated patients was 66% at 1 year and 59% at 5 years. In operated patients, none had a relapse, although six developed late recurrence. Freedom from infection, re-operation and long-term mortality were similar in patients undergoing AVR compared to ARR.

**Conclusion:**

Patients diagnosed with intra-cardiac abscess who were not operated on had very poor survival. In those who underwent an operation, either by AVR or ARR based upon patient factors, imaging and intra-operative findings outcomes were similar.

## Graphical Abstract

Choice of surgical technique was not associated with survival, re-infection or re-operation. 
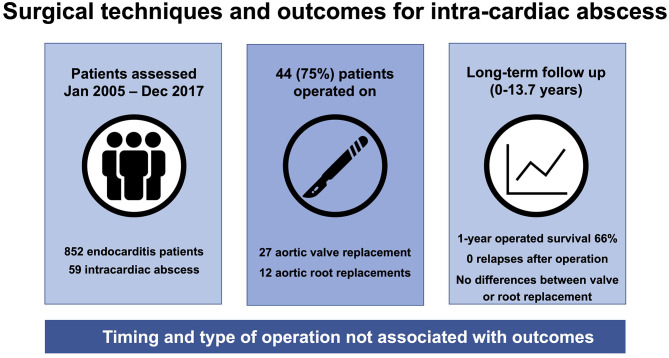

## Introduction

### Background

An intra-cardiac abscess is a serious complication of both native (NV-IE) and prosthetic valve infective endocarditis (PV-IE), with a reported prevalence of 14.4% (1). The risk of development of intra-cardiac abscesses is greater with virulent pathogens, particularly *S. aureus*, which is more common in PV-IE and usually affects the aortic valve (2, 3). Antibiotic therapy alone is rarely adequate, and when untreated, this complication is usually fatal as a consequence of valvular dysfunction, fistula formation, pseudoaneurysm, or arrhythmia due to the involvement of the atrioventricular node (4).

Intra-cardiac root abscesses can be managed by repair of the peri-annular root abscess and aortic valve replacement (AVR) or reconstruction of the aortic root or aortic root replacement (ARR) (2, 5). While intra-cardiac abscess is an accepted indication for surgery according to current guidelines, the optimal timing and type of operation is unknown (6). Whether the diagnosis should prompt an immediate operation to achieve source control and prevent further destruction of native tissues, or whether there should be a period of antibiotic therapy to achieve clinical stabilization prior to undertaking surgery is unknown. Further, it is unknown whether abscesses affecting the aortic root are best managed by AVR or ARR.

### Aim

We aimed to evaluate the influence of timing and choice of operation on outcomes in patients with intra-cardiac abscesses presenting over more than a decade.

## Patients and Methods

### Study Design

This is a retrospective, observational study that is designed and reported according to the Strengthening the Reporting of Observational Studies in Epidemiology (STROBE) statement (7).

### Setting

The study was conducted at the Leeds Teaching Hospitals NHS Trust, a high-volume and tertiary referral center for cardiology and cardiac surgery.

### Participants

Consecutive patients aged ≥18 years managed for intra-cardiac abscess between 1 January 2005 and 31 December 2017 were eligible to be included in the study and were identified from a prospectively maintained IE database. We required episodes to be definite, according to the modified Duke criteria (8), with intra-cardiac abscess confirmed by pre-operative cardiac imaging or at operation. All the patients were referred to the multidisciplinary IE team for consideration of surgery, and decisions regarding operation were made by consensus consistent with the international guidelines (6, 9).

### Variables, Data Sources and Outcomes

Clinical data were obtained from the Leeds Patient Pathway Manager Plus electronic healthcare record and written medical records. Vital status data were collected using linked Office of National Statistics mortality data with final censorship occurring in May 2019. During the study period, changes in pre-operative risk scoring occurred [following the publication of the logistic Euroscore (10) and Euroscore II (11)], and to ensure consistency, we retrospectively calculated Euroscore II for all operated patients. Dates and causes of death were recorded, and where necessary, this information was obtained by request to the Coroner's office.

### Bias

Consecutive patients were identified from a prospectively collected IE database, and to ensure complete case ascertainment, additional searches of both echocardiography and surgical databases for the term ‘abscess' were performed and manually evaluated. The variables, outcomes and analyses were specified prior to the data collection, and no imputation for missing data was made.

### Definitions

Intra-cardiac abscess was defined as either a true abscess, a closed cavity containing infected material, or infected aortic root aneurysms in the context of Duke definite IE. The microbial cause of abscess was determined by blood culture and analysis of tissue from excised valves obtained at operation. Surgery was classified according to guideline recommendations as an emergency if performed ≤24 h following diagnosis of intra-cardiac abscess, urgent if ≤7 days and elective if ≥7 days but undertaken during the index episode in patients who were receiving antibiotics at the time of operation (6). We differentiated between re-infection caused by the same pathogen occurring within 1 year of the index episode, termed “relapse”, and a further episode of IE caused by a different pathogen or occurring beyond 1 year, termed “recurrence”, as previously published (12). The start of an episode was either the date of first blood culture sampling or a diagnostic echocardiogram.

### Sample Size

This was an observational study, and the sample size was not pre-specified.

### Outcomes

The primary outcome was all-cause 30-day surgical mortality. Secondary outcomes were freedom from re-infection, re-operation and long-term all-cause mortality in patients with aortic root abscess undergoing AVR compared to ARR.

### Statistical Analysis

All statistical analyses were performed using IBM SPSS Statistics version 26 (IBM Corporation, Armonk, NY). Continuous variables are presented as median [interquartile range (IQR)] and discrete variables as number (percentage). Groups were compared by the Mann–Whitney U test for continuous data and by Pearson χ^2^ tests for categorical data. Unadjusted survival analysis is displayed by Kaplan–Meier plot with survival difference determined by the log-rank test. In all analyses, statistical significance was defined as *p* < 0.05.

### Ethical Considerations

Approval was given following an institutional governance review (IRB approval 16/09/2019 #7562). In view of the retrospective nature and the presentation of cohort data rather than individual patient identifiers, individual patient consent was waived. Appropriate data protection policies were followed throughout the analysis.

## Results

### Participants

During the study period, a total of 852 patients who had Duke definite IE were reviewed by the IE team at our institution, of whom 59 (6.9%) developed an intra-cardiac abscess. Table 1 presents the clinical characteristics of patients with intra-cardiac abscess who had a median age of 55 (43–71) years, of whom 44 (75%) were men and 10 (17%) were persons who injected drugs (PWID). Twenty-six (44%) patients initially presented to our center, and the remaining presented to local hospitals and were transferred for ongoing care. The median hospital stay for all the patients was 49 (33–66) days, and for operated patients, the length of the stay in the intensive care unit (ICU) was 5 (3–8) days.

**Table 1:** Characteristics of patients with intra-cardiac abscess who were operated on or received antibiotics alone.

**Variable**	**All**	**Operated**	**Not operated**	***p*-value**
	**(*n =* 59)**	**(*n =* 44)**	**(*n =* 15)**	
Age (years)	55 (43–71)	52.5 (41–65.8)	71 (59–78)	0.004
Male sex [*n* (%)]	44 (75)	33 (75)	11 (73)	0.9
PWID [*n* (%)]	10 (17)	9 (20)	1 (7)	0.22
PV-IE [*n* (%)]	28 (47)	18 (31)	10 (67)	0.08
*S. aureus* [*n* (%)]	8 (14)	6 (14)	2 (13)	0.98
Multiple valves affected [*n* (%)]	14 (24)	9 (20)	5 (33)	0.31
Fistula [*n* (%)]	5 (8)	2 (5)	3 (20)	0.07
BCAV [*n* (%)]	8 (14)	8 (18)	0 (0)	0.08
LVEF (%)	55 (45–55)	55 (35–55)	55 (45–55)	0.61
Heart failure [*n* (%)]	41 (69)	29 (66)	12 (80)	0.31
Heart block/pacing [*n* (%)]	12 (20)	12 (27)	0 (0)	0.02
Pre-versusoperative stroke [*n* (%)]	9 (15)	7 (16)	2 (13)	0.81
Pulmonary embolus [*n* (%)]	2 (3)	2 (5)	0 (0)	0.4
Splenic abscess [*n* (%)]	7 (12)	6 (14)	1 (7)	0.47
Other emboli [*n* (%)]	10 (17)	9 (20)	1 (7)	0.22
AKI [*n* (%)]	11 (19)	7 (16)	4 (27)	0.36
Septic arthritis [*n* (%)]	3 (5)	2 (5)	1 (7)	0.75
Interval to diagnostic imaging (days)	8 (4–16)	7.5 (4–15)	15 (5.5–31.5)	0.19
Hospital stay (days)	49 (33–66)	49.5 (33–66)	47 (19–64)	0.82

*PWID, person who injects drugs; PV-IE, prosthetic valve infective endocarditis; BCAV, bicuspid aortic valve; LVEF, left ventricular ejection fraction; AKI, acute kidney injury*.

### Location of Abscess and Affected Structures

The location of the intra-cardiac abscess was usually the aortic root, although it was confined to the anterior mitral valve annulus in two patients and posterior mitral valve annulus in four patients, with one abscess involving the posterior mitral valve annulus and interventricular septum. Affected valves were primarily the aortic (55) and mitral (17) valves, with one instance of tricuspid valve infection. Thirty-one (53%) had NV-IE and 28 (47%) had PV-IE, of which 10 (17%) occurred within 1 year of operation. Eight (14%) patients with NV-IE had a bicuspid aortic valve and one (1%) had a quadricuspid valve. Cusp involvement for patients with aortic root abscess undergoing AVR and ARR is presented in Table 2, involving multiple cusps in 28 (72%) patients.

**Table 2:** Operative data for patients with aortic root abscess undergoing AVR and ARR.

**Variable**	**All**	**AVR**	**ARR**	***p*-value**
	**(*n =* 39)**	**(*n =* 27)**	**(*n =* 12)**	
Location of abscess	24 (62)	17 (63)	7 (58)	0.78
NCC [*n* (%)]				
LCC [*n* (%)]	23 (59)	17 (63)	6 (50)	0.45
RCC [*n* (%)]	25 (64)	18 (67)	7 (58)	0.62
Multiple cusps [*n* (%)]	28 (72)	20 (74)	8 (67)	0.64
Euroscore II	9.0 (3.8–15.2)	5.0 (2.8–12.4)	14.3 (11.2–23.7)	0.002
**ECG**	27 (69)	18 (67)	9 (75)	0.6
SR [*n* (%)]				
Paced [*n* (%)]	12 (31)	9 (33)	3 (25)	0.6
CC time	106 (74.5–172.8)	94 (74–110)	213 (123.3–271.5)	<0.001
CPB time	150 (107–252.3)	126 (101–169)	523 (239.5–378.8)	<0.001
Re-operation for bleeding [*n* (%)]	6 (15)	3 (11)	3 (25)	0.11
ICU stay (days)	5 (3–8)	4 (3–8)	6 (4.3–11)	0.38
Hospital stay (days)	49 (33–66)	46 (27–57)	64 (51–72.8)	0.049

*AVR, aortic valve replacement; ARR, aortic root replacement; NCC, non-coronary cusp; LCC, left coronary cusp; RCC, right coronary cusp; AMVL, anterior mitral valve leaflet; PMVL, posterior mitral valve leaflet; SR, sinus rhythm; CC, cross-clamp; CPB, cardiopulmonary bypass; ICU, intensive care unit*.

### Microbiology

The microorganisms causing aortic root abscess were primarily *Staphylococcus* (20) and *Streptococcus* (27) species. There were mixed infections in two patients, with each having two microorganisms (in both a combination of *Staphylococcus* and *Streptococcus* species), and one patient was blood culture negative. Infection with coagulase-negative staphylococci was more common in PV-IE (*p* = 0.005), while infection with beta-haemolytic streptococci was more common in NV-IE (*p* = 0.009). Early PV-IE (within 1 year) was usually caused by coagulase-negative staphylococci (seven), but also by *S. aureus* (one), *Candida* (one) and *Corynebacterium* species (one).

### Cardiac Imaging

There were a total of 121 cardiac imaging data sets, 72 of which were transthoracic (TTE) and 69 were transoesophageal (TOE) echocardiograms, three were cardiac computerized tomography and one was magnetic resonance imaging. The initial diagnosis of the abscess was made by TOE in 33 (56%) episodes and by TTE in 24 (41%) episodes, and for two patients, cardiac imaging data were missing. The median interval between first blood culture sampling and diagnostic imaging was 8 (4–16) days and was not different between operated and non-operated patients (*p* = 0.19) (Table 1).

### Additional Complications of IE

The most common additional complication was heart failure, which occurred in 40 (68%) episodes. Stroke occurred in nine (15%), splenic emboli in seven (12%), pulmonary emboli in two (3%) and other emboli in eight (9%) episodes. Acute kidney injury complicated eight (14%), septic arthritis complicated three (8%) and heart block or need for permanent pacing complicated 12 (20%) episodes. In all cases of heart block, the aortic valve was affected, and eight of these had extensive abscess formation involving two or more sinuses.

### Surgery

**Figure 1:**
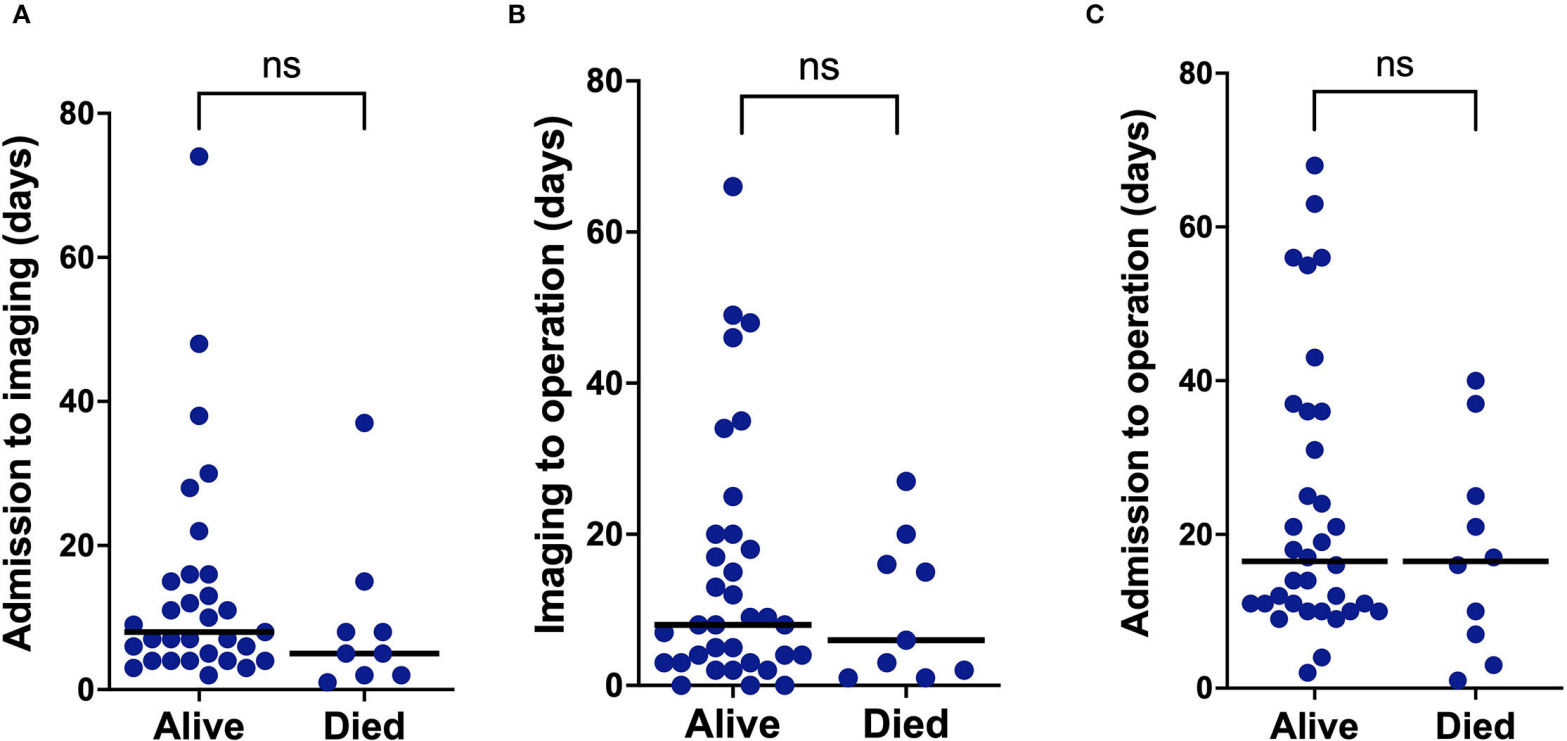
Plots of the time interval between **(A)** start of the episode to diagnostic imaging, **(B)** time from diagnostic imaging to operation, and **(C)** time from the start of the episode to operation, comparing patients who were alive or had died within 30 days of operation.

Forty-four (75%) patients were operated on during their index episode and 15 (25%) were not. The median time between diagnosis of intra-cardiac abscess and operation was 8 (3–20) days. Three emergency operations were undertaken (≤24 hours), 17 patients were operated on urgently (≤7 days) and the remaining 24 patients underwent elective (>7 days) operations. Additionally, one patient underwent surgery following relapse, having received conservative management initially. At operation, an abscess was confirmed in all the cases, and in three cases, there was evidence of a healed abscess. Operated patients were younger [52.5 (41–65.8) vs. 71 (59–78) years; *p* = 0.004] and more likely to have developed heart block or required permanent pacing (27% vs. 0%; *p* = 0.02) (Table 1). The presence of abscess complicated by heart failure was the documented primary indication for operation in 29 (67%), uncomplicated aortic root abscess in 13 (30%) and failure of medical therapy in two (3%) patients. In comparing the patients who were alive or had died within 30 days of operation, there were no differences in the interval between admission and diagnostic imaging [8 (4–16) vs. 5 (2–11.5) days; *p* = 0.19], the time between diagnostic imaging and operation [8 (3–20) vs. 6 (1.5–18) days; *p* = 0.53] or the time between admission and operation [17.5 (10–34.8) vs. 16.5 (6–27.5) days; *p* = 0.38] (Figure 1). Additionally, the interval between diagnostic imaging and operation was not different between patients presenting to our center or those referred from the district hospitals (*p* = 0.79).

Twenty-seven patients underwent AVR with or without patching of abscess cavity, and 12 underwent ARR with composite (12) or interposition grafts (two). Two patients underwent isolated mitral valve replacement (MVR), and one underwent MVR with patching of the abscess cavity. The three patients with healed abscesses underwent AVR with simultaneous patching of the abscess cavity. Patients with PV-IE underwent ARR more often, and this was performed in 10 (77%) compared to eight (29%) patients with NV-IE (*p* = 0.004). Cross-clamp (CC) and cardiopulmonary bypass (CPB) times were not different in patients who were alive or dead 30 days following surgery but were significantly longer in patients undergoing ARR compared to AVR (*p* < 0.001). Time in ICU was not different between groups, although hospital stay was longer in patients undergoing ARR (*p* = 0.049). Re-operation for bleeding occurred in three patients undergoing AVR (11%) and three undergoing ARR (25%) (*p* = 0.27). The post-operative stroke did not occur in any patient. Of the 15 patients who did not undergo an operation during their index episode, underlying reasons included prohibitively high pre-operative risk in 12 and death prior to surgery in three. Two patients managed conservatively were taken care of in ICU prior to death.

### Relapse and Recurrence

One episode of relapse occurred following conservative management during the index episode, with this individual subsequently undergoing surgery. There were no relapses in the operated patients, although recurrence occurred following a median interval of 2,231 (248–2,757) days in six patients. Three patients developed recurrence with a different pathogen within 1 year; in one case, this was due to *S. aureus* infection in the context of PWID, and the other cases were caused by *Enterococcus* spp. and beta-haemolytic streptococci. The three late recurrences were caused by *Enterococcus* spp., oral *Streptococcus* spp. and *S. anginosus*.

### Survival

**Figure 2:**
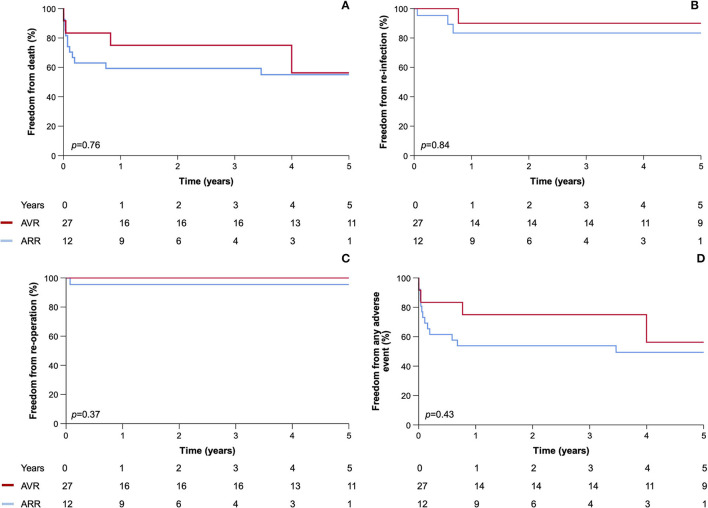
Kaplan–Meier plots of **(A)** all-cause mortality, **(B)** relapse/recurrence, **(C)** re-operation, and **(D)** any adverse event comparing patients with aortic root abscess undergoing AVR and ARR.

**Figure 3:**
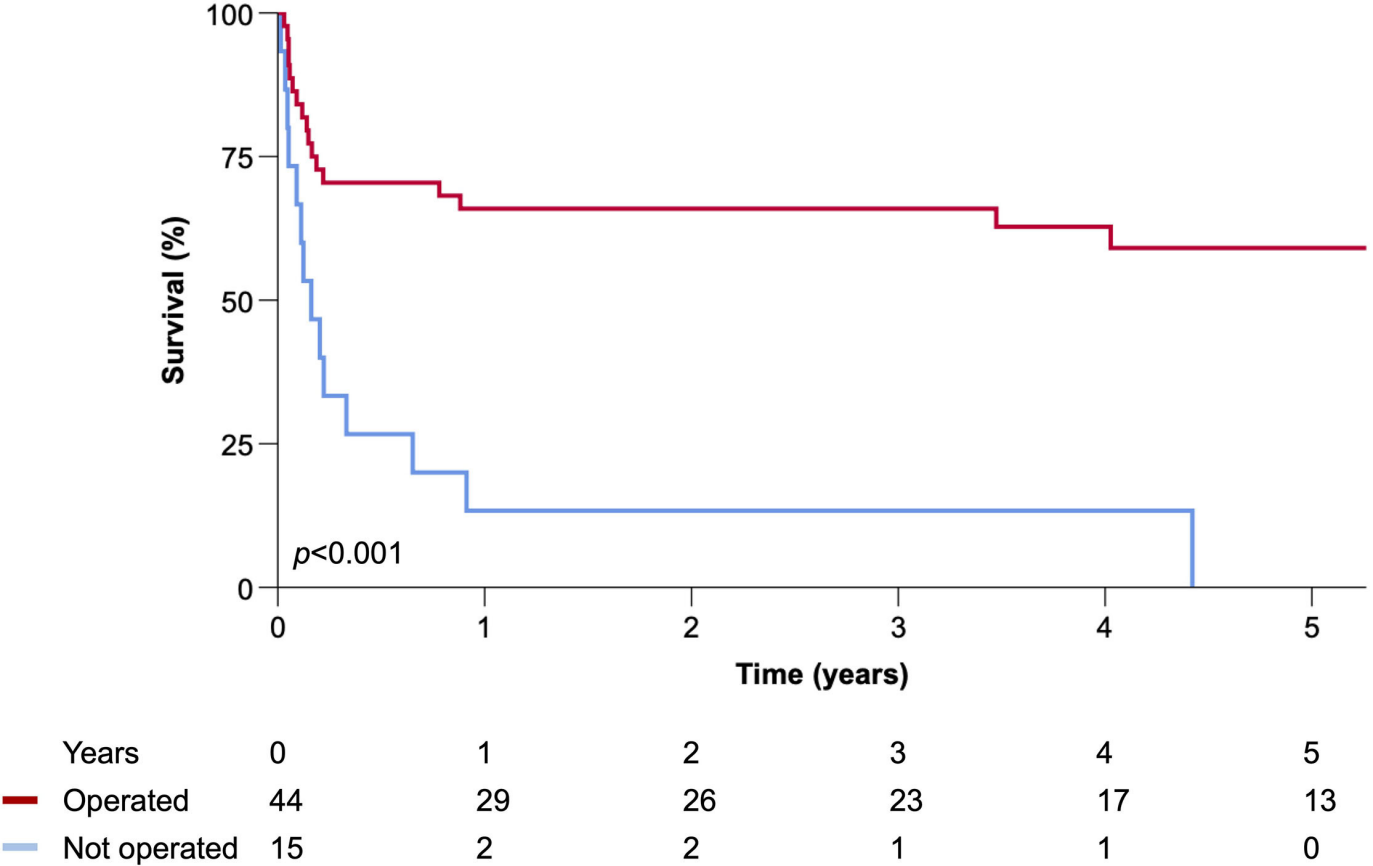
Kaplan–Meier plot of all-cause mortality comparing operated and non-operated patients.

During a median follow-up of 1.9 (range 0–13.7) years, there were 35 (59%) deaths. The 30-day surgical mortality rate (17%) was associated with infection due to *S. aureus* (6% vs. 40%; *p* = 0.006) with a trend toward higher Euroscore II in those who died [8.0 (3.6–12.6) vs. 15.4 (45–32.0); *p* = 0.12] (Table 3). Survival in operated patients was 66% at 1 year and 59% at 5 years. Freedom from relapse/recurrence, re-operation, all-cause mortality and a composite of all adverse events were not different in patients undergoing AVR when compared to those undergoing ARR (Figure 2). All 15 patients who did not undergo an operation died during follow-up [median time 59 (19–238) days], although one survived their index episode and lived for a further 2.3 years with an apparent cure on serial cardiac imaging follow-up (Figure 3).

**Table 3:** Characteristics of operated patients who were alive or dead at 30 days.

**Variable**	**All**	**Alive**	**Dead**	***P*-value**
	**(*n =* 44)**	**(*n =* 34)**	**(*n =* 10)**	
Age (years)	55 (43–71)	52.5 (40.3–65.3)	51.5 (40–75)	0.73
Male sex [*n* (%)]	33 (75)	26 (76)	7 (70)	0.68
PWID [*n* (%)]	9 (20)	6 (18)	3 (30)	0.4
PV-IE [*n* (%)]	18 (41)	14 (41)	4 (40)	0.95
*S. aureus* [*n* (%)]	6 (14)	2 (6)	4 (40)	0.006
Multiple valves affected [*n* (%)]	9 (20)	6 (18)	3 (30)	0.4
Multiple sinuses [*n* (%)]	28 (64)	22 (65)	6 (60)	0.79
BCAV [*n* (%)]	8 (18)	7 (21)	1 (10)	0.45
Euroscore II	9.0 (3.8–15.2)	8.0 (3.6–12.6)	15.4 (4.5–32.0)	0.12
Interval from start of episode (days)	17 (10–33.5)	17.5 (10–34.8)	16.5 (6–27.5)	0.38
Interval from diagnostic imaging (days)	8 (3–20)	8 (3–20)	6 (1.5–18)	0.53
LVEF (%)	55 (45–55)	55 (45–55)	45 (35–55)	0.14
ARR [*n* (%)]	13 (30)	11 (32)	2 (20)	0.49
CABG [*n* (%)]	8 (18)	6 (18)	2 (20)	0.87
CC time (mins)	106 (74.5–172.8)	101.5 (74–186.5)	111 (86.8–157.5)	0.84
CPB time (mins)	150 (107–252.3)	136 (107–300)	171.5 (136–247)	0.88
Time on ICU (days)	5 (3–8.5)	5 (3–75)	10 (2.5–15)	0.22

*PWID, person who injects drugs; PV-IE, prosthetic valve infective endocarditis; BCAV, bicuspid aortic valve; LVEF, left ventricular ejection fraction; ARR, aortic root replacement; CABG, coronary artery bypass grafting; CC, cross-clamp; CPB, cardiopulmonary bypass; ICU, intensive care unit*.

### Causes of Death

Medical certificates for the cause of death were available for 34 of the 35 patients who died during follow-up (97%). The causes of death were consequent to IE and its complications in 31 (89%) patients, which was usually due to congestive cardiac failure (19) but also uncontrolled infection (three), of which one was an early recurrent infection caused by *S. aureus*. Other causes were thromboembolism causing mesenteric ischaemia (four) or stroke (one), death during surgery (two) or sudden cardiorespiratory arrest (two). In the remaining patients, mortality was caused by malignancy (two) in the conservatively managed patients who survived their index episode, with one death due to methadone overdose and one cause of death unascertained.

## Discussion

In this study, we have reported the surgical management, complications and long-term outcomes in patients managed for intra-cardiac abscesses over more than a decade. We found that intra-cardiac abscess was an uncommon complication of IE, with a majority of the patients undergoing an operation, and that the all-cause mortality rate in the short and long term was high. Without an operation, intra-cardiac abscess was almost universally fatal, although the type or timing of the operation was not associated with the outcomes.

### Patient Demographics and the Diagnosis of Intra-Cardiac Abscess

The demographic characteristics of the patients were broadly similar to those reported in the earlier studies, with the majority being men and of relatively younger average age compared to IE in general (1). However, we observed that the proportion of PWID was 17%, approximately twice that expected based on the demographics of patients recruited to registry studies, (1) but similar to other studies of IE complicated by intra-cardiac abscess, (13) suggesting this might be an important aetiological factor for its development. Patients presenting with IE and who are PWID, therefore, require careful evaluation for this particular complication, which may be a consequence of delayed presentation or more virulent pathogens (12). Transthoracic echocardiography was often sufficient to diagnose an intra-cardiac abscess, although most patients underwent transoesophageal echocardiography prior to operation. The median time between the start of an episode and diagnostic imaging was 8 (4–16) days, which is longer than the ideal period, but reflects the time until imaging was diagnostic of IE, rather than the first imaging study that was undertaken. It is possible that delays in care contributed to the development of an abscess in some who were cared for initially in non-specialist settings or other centers prior to referral.

### Operative Technique and the Association With Outcomes

Surgical management of intra-cardiac abscess involves debridement and removal of infected tissue, exclusion of the abscess cavity from the circulation and replacement of the infected valve (14). A recent meta-analysis comparing ARR and AVR showed a significant reduction in re-operation in patients undergoing ARR (relative risk 0.50, 95% CI 0.26–0.94), (4) but this was not replicated in our analysis, where the surgical approach was not associated with survival, relapse/recurrence or re-operation. As expected, the CPB and CC times were significantly longer in patients undergoing ARR, but this was not associated with worse outcomes, although hospital stays were longer. Importantly, there was no adverse safety signal associated with ARR compared to AVR, with similar rates of re-operation for bleeding. This was despite a higher proportion having PV-IE with more extensive destruction of native tissue and on average a higher Euroscope II, although in part this was due to the greater weighting of the intervention for patients undergoing ARR. Several groups have advocated the use of allogenic materials, but the results in published studies are variable, and while some report exclusively on outcomes following ARR, some include patients undergoing both AVR and ARR (15–17). In our study, few patients underwent ARR using allogenic materials, so a comparison was not possible.

### Microbial Etiology and Risk Factors for Development of Intra-Cardiac Abscess

In the earlier studies, the microbiological diagnosis was often not reported, and in others, the culture-negative rate was as high as 32.7% (18). In contrast, the causative pathogen was identified in 98% of our patients, and a targeted antibiotic strategy was possible for nearly all patients. The use of non-specific antibiotic regimens might be expected to be associated with inadequate treatment and more extensive tissue destruction, and it is possible that the earlier diagnosis and initiation of therapy supported by a dedicated IE team in our institution may account for the relatively lower rate of intra-cardiac abscess observed (1). However, once an intra-cardiac abscess has developed, our findings indicate that directed antibiotic therapy does not mitigate the need for operation and that the failure of medical therapy does not influence the decision to operate. As expected, we observed that NV-IE was more often associated with streptococci and PV-IE with coagulase-negative staphylococci, which was observed frequently in early PV-IE (occurring within 1 year), suggesting that nosocomial introduction of virulent pathogens at the time of surgery may be an aetiological factor in the development of the intra-cardiac abscess in this group.

Previous studies have reported increased mortality rates in those with abscesses caused by *S. aureus* and in patients with PV-IE (19, 20). Consistent with these observations, we found that patients with *S. aureus* infection displayed higher 30-day surgical mortality, although no other variables were associated with this outcome, including the timing and type of operation undertaken. There were no relapses in the operated patients; hence, although the need for surgery is not affected by appropriate antibiotic therapy, it probably affects the risk of relapse. A significant number of recurrences were observed in the operated patients with most of them occurring late, although in one instance, early recurrence was caused by *S. aureus* in a patient who continued to inject drugs, reflecting the ongoing risk of infection in these patients following complex cardiac surgery. Potential risk factors for intra-cardiac abscess formation in these patients include the large surface area of prosthetic materials and the high proportion of subjects who were PWID. In this study, PWID developed left-sided IE, reflecting the inclusion of patients with intra-cardiac abscess, although IE in PWID is often left-sided (12).

### Decision for and Timing of Surgery

Although our strategy was to perform surgery in all suitable cases, the decision not to operate in around a quarter of patients due to prohibitively high pre-operative risk is consistent with other reports (21). The reasons for not opting for an operation were usually prohibitively high surgical risk; however, we were unable to determine Euroscore II in non-operated patients, as the urgency, status of the patient and type of operation undertaken cannot be determined retrospectively in non-operated patients. Patients managed conservatively usually did not survive, although an apparent cure was achieved for one patient who received a prolonged course of antibiotic therapy, which although rare has been reported in the literature (22, 23). In fact, we found healed abscess at operation in three instances, suggesting resolution, albeit in the context of aortic valve IE causing heart failure necessitating AVR.

While some studies describe a preference for early surgery (17, 24), few have described the timing of operation relative to the diagnosis (25, 26). Higher 30-day surgical mortality rate following emergency surgery has been reported (15), but this was not confirmed in a pooled analysis (4). Few patients underwent surgery on an emergency basis (≤24 h), due to a local preference for achieving clinical stabilization prior to operation, although surgery was on average undertaken earlier than in previous reports. As a continuous variable, the timing of operation was not associated with 30-day survival, although given the low number of patients, there is a risk that this may represent a type two error.

### Long-Term Outcomes

The high mortality rate in the long term for operated patients despite a relatively young average age is concerning and is found to be higher than the rate reported previously (2, 27), although must be balanced against the small number of patients at risk at 5 years. While two patients died of cancer and one of methadone overdose, the majority died from IE and its complications, either during the index episode or due to relapse or recurrence. Previous studies of surgery for IE not associated with abscess formation and following surgery for intra-cardiac abscess are broadly similar, showing a poor long-term survival rate (28).

### Strengths and Limitations

This was a retrospective study including a modest number of cases and conducted at a single center, where the decision about the timing and the type of operation was made on an individual basis at the discretion of the operating surgeon. Our data are therefore susceptible to measured and unmeasured confounders, such as clinical status of operated patients and extent of tissue destruction, which may have influenced outcomes beyond the choice of operative technique. The prevalence of intra-cardiac abscess was lower than has been reported, possibly owing to a low rate of culture negativity enabling targeted antibiotic therapy. In addition, by reporting only Duke definite IE, it is likely that we have overestimated the prevalence of abscesses in our patients.

## Conclusion

These data support an individualized approach for intra-cardiac abscess management, synthesizing the clinical status, pre-operative imaging and direct inspection of the surgical field. Guidelines advocate more urgent surgery in this setting, and although our results do not support this, it is feasible that for non-operated patients who died while awaiting an operation, more prompt surgery may have been advantageous. Additional findings were that PV-IE usually required ARR, relapse rates were low following surgery and intravenous drug use is an important aetiological factor. Finally, although intra-cardiac abscess has a dismal prognosis without surgery, around a quarter of patients were not surgical candidates due to prohibitively high pre-operative risk.

## Data Availability Statement

The data analyzed in this study is subject to the following licenses/restrictions: Data are available from the corresponding author upon reasonable request. Requests to access these datasets should be directed to s.straw@leeds.ac.uk.

## Ethics Statement

The studies involving human participants were reviewed and approved by Clinical Governance Lead, Leeds Teaching Hospitals NHS Trust. Written informed consent for participation was not required for this study in accordance with the national legislation and the institutional requirements.

## Author Contributions

VM, MB, and JS researched the topic and devised the study. VM, MB, RG, and JS collected the data. SS analyzed the data. SS and MB produced the first draft of the manuscript. All authors provided critical revision of the manuscript.

## Funding

This study was supported by the British Heart Foundation (FS/CRTF/20/24071).

## Conflict of Interest

JS has participated in research funded by Pfizer and Merck Sharpe and Dohme. KW has received speakers' fees and honoraria from Medtronic, Cardiac Dimensions, Novartis, Abbott, BMS, Pfizer, and Bayer has received an unconditional research grant from Medtronic. The remaining authors declare that the research was conducted in the absence of any commercial or financial relationships that could be construed as a potential conflict of interest.

## Publisher's Note

All claims expressed in this article are solely those of the authors and do not necessarily represent those of their affiliated organizations, or those of the publisher, the editors and the reviewers. Any product that may be evaluated in this article, or claim that may be made by its manufacturer, is not guaranteed or endorsed by the publisher.

## Abbreviations

NV-IE: native valve infective endocarditis
PV-IE: prosthetic valve infective endocarditis
AVR: aortic valve replacement
ARR: aortic root replacement
IQR: interquartile range
PWID: person who injects drugs
ICU: intensive care unit
TTE: transthoracic echocardiography
TOE: transoesophageal echocardiography
MVR: mitral valve replacement
CC: cross-clamp
CPB: cardiopulmonary bypass.
